# The Prevalence of Anti-Zein Antibodies: A Comparative Study between Celiac Disease and Irritable Bowel Syndrome

**DOI:** 10.3390/nu13020649

**Published:** 2021-02-17

**Authors:** Luis Alberto Sánchez-Vargas, Karina Guadalupe Hernández-Flores, Francisco Javier Cabrera-Jorge, José María Remes-Troche, Job Reyes-Huerta, Héctor Vivanco-Cid

**Affiliations:** Instituto de Investigaciones Médico Biológicas, Universidad Veracruzana, Calle Agustín de Iturbide, SN, 91700 Veracruz, Mexico; kimicoluis@hotmail.com (L.A.S.-V.); karinhernandez@uv.mx (K.G.H.-F.); Fco-Javier-CJ@hotmail.com (F.J.C.-J.); joremes@uv.mx (J.M.R.-T.); jobnet1@hotmail.com (J.R.-H.)

**Keywords:** celiac disease, irritable bowel syndrome, anti-zein antibodies, humoral immune response

## Abstract

Celiac disease (CD) is a chronic immune-mediated enteropathy triggered by exposure to dietary gluten in genetically predisposed individuals. In contrast, irritable bowel syndrome (IBS) is a common functional gastrointestinal disorder affecting the large intestine, without an autoimmune component. Here, we evaluated the prevalence of IgA and IgG antibodies to maize zeins (AZA) in patients with CD and IBS. Using an in-house ELISA assay, the IgA and IgG anti-zein antibodies in the serum of 37 newly diagnosed CD (16 biopsy proved and 21 serological diagnosis) and 375 IBS patients or 302 healthy control (HC) subjects were measured. Elevated levels of IgA AZA were found in CD patients compared with IBS patients (*p* < 0.01) and HC (*p* < 0.05). CD patients had the highest prevalence (35.1%), followed by IBS (4.3%) and HCs (2.3%) (*p* < 0.0001). IgG AZA antibodies were not found in any CD patients, IBS patients, or HC subjects. A significant positive correlation was found between IgA AZA with IgA anti-gliadin (AGA, *r* = 0.34, *p* < 0.01) and IgA anti-deaminated gliadin peptides (DGP, *r* = 0.42, *p* < 0.001) in the celiac disease group. Taken together, our results show for the first time a higher prevalence of AZA IgA antibodies in newly diagnosed CD patients than in IBS patients, confirming a biased immune response to other gliadin-related prolamins such as maize zeins in genetically susceptible individuals.

## 1. Introduction

Celiac disease (CD) is a systemic autoimmune disorder that not exclusively affects the small intestine [1,2,3,4] in genetically susceptible individuals (HLA DQ2 and/or DQ8 haplotypes). CD is caused by an abnormal intestinal immune response to proline- and glutamine-rich gluten proteins from the triticeae tribe of the Gramineae family (wheat, rye, and barley) [1]. Oats can induce some clinical manifestations in a small fraction of CD patients, even though they are distantly related to wheat [5,6]. Rice, maize, sorghum, and millet are still more distantly and less related cereals to wheat, and they have been considered nontoxic to CD patients [7]; however, recent studies seem to show that some of these cereals such as maize, traditionally considered safe for celiac patients, could activate the immune response in some celiac patients [8]. Although wheat consumption in Mexico is common, maize is the most important cereal in the Mexican diet and is a common substitute to wheat used in the gluten-free diet (GFD) of CD patients. CD affects about 0.59% of the Mexican population, a figure which is similar to data reported for other populations in the American continent [9,10].

On the other hand, a recent meta-analysis reported that CD is four-fold more prevalent in patients with a clinical presentation of irritable bowel syndrome (IBS) versus non-IBS populations [11]. Both IBS and CD have overlapping clinical symptoms and can be confused with one another. IBS is a clinical syndrome defined, according to the Rome III criteria, by the presence of abdominal pain or discomfort at least 3 days per month and two other symptoms related to stool frequency and appearance [12]. IBS is the most important functional gastrointestinal disorder in Mexico, with a prevalence of around 16% [11]. Food intolerance is a common complaint amongst patients with IBS. There are many reports of the food-induced aggravation of IBS and improvement after the dietary exclusion of food based on the serum IgG antibody response [13,14,15,16,17]. Interestingly, some common foods have been reported as symptom triggers in IBS patients, including grains such as wheat, barley, rye, oats, and maize [18,19]. An immune response to specific dietary constituents, such as gluten or other prolamins, may be responsible for the generation of IBS-like symptoms in some susceptible individuals. Whereas the prevalence of anti-gluten antibodies in CD and IBS patients has been described before [10,20], studies regarding the humoral immune response (specific IgA and IgG antibodies) to maize prolamins in CD and IBS patients are lacking. Therefore, the present study determined and compared the prevalence of IgA and IgG anti-zein antibodies in a group of Mexican patients diagnosed with CD or IBS and healthy controls.

## 2. Materials and Methods

### 2.1. Study Populations

This study was carried out at the Laboratorio Multidisciplinario en Ciencias Biomédicas and the Laboratorio de Fisiología Digestiva y Motilidad Gastrointestinal at the Instituto de Investigaciones Medico-Biológicas, Universidad Veracruzana (Veracruz, México). A total of 714 serum samples were included from December 2010 to May 2013. All the serum samples were frozen at −80 °C until the time of analysis. Thirty-seven newly diagnosed CD patients were included. In all cases, serum samples were positive for CD-specific antibodies (IgA anti-tissue transglutaminase (tTG), IgA and IgG anti-deaminated gliadin peptide (DGP)). Subjects with only positive anti-DGP antibodies were not included. Serum samples were analyzed for IgA tTG, IgA/IgG DGP, and IgA/IgG anti-native gliadin (IgA/IgG AGA) using commercial assays for IgA h-tTG (QUANTA Lite, INOVA Diagnostics Inc., San Diego, CA, USA), IgA/IgG DGP ELISA, and IgA/IgG Gliadin ELISA (INOVA Diagnostic Inc., San Diego, CA, USA). All the antibodies were tested at the time of the diagnosis and before a gluten free diet (GFD). Assays were carried out in accordance with the manufacturer’s instructions. Negative values were setup at < 20 U/mL, the weakly-positive value was set up to be 20–30 U/mL, and the positive value was > 30 U/mL according to the cut-off values recommended by the manufacturer. Sixteen subjects agreed to undergo duodenal biopsy to confirm the CD diagnosis. The Marsh–Oberhüber classification was used to assess the histological damage [21]. In the 16 subjects in whom the diagnosis of CD was confirmed by duodenal biopsy, the distribution according to the Marsh–Oberhüber classification was as follows: grade II 31% (5/16), grade IIIa 50% (8/16) and grade IIIb 19% (3/16). Regarding the IBS group, 85% (*n* = 320) were female (mean age 43 years, Table 1) and IBS with mixed pattern (IBS-M) was the most common subtype (56%, *n* = 210), followed by IBS with constipation (IBS-C) (32%, *n*= 121) and IBS with diarrhea (IBS-D) (12%, *n* = 44). The diagnosis and categorization of IBS patients was based on the Rome III criteria, and only those with IBS without CD (excluded by the absence of CD-specific antibodies) were included in the IBS group. Patients with IBS were divided into three subgroups based on stool form: IBS with constipation, IBS with diarrhea, and mixed profile IBS. The control group was made up of asymptomatic healthy controls (in accordance with physical examination and clinical evaluation). These subjects were recruited from an open population (same geographical region) and their samples had been previously collected for population studies in the Veracruz-Boca del Río region at Centro Estatal de la Transfusión Sanguínea of Veracruz. All the healthy controls were negative for CD-specific antibodies and answered the Rome III questionnaire to confirm the absence of gastrointestinal symptoms.

### 2.2. Ethics Statement

Written consent to participate in the study was obtained from each participant after a full explanation of the study in accordance with the guidelines of the Ethics Committee of the Instituto de Investigaciones Medico-Biológicas, Universidad Veracruzana, number IIMB-20-010-14.

### 2.3. In-House Enzyme-Linked Immunosorbent Assay (ELISA) for IgA/IgG AZA

All the technical procedures were carried out at room temperature. Briefly, plates were coated with 100 µL of zein (Sigma-Aldrich, St. Louis, MO, USA) at 10 µg/mL in 70% ethanol for 1 h. The plates were then washed three times with phosphate-buffered saline (PBS) at pH 7.4 containing 0.05% Tween-20 (PBST). Residual protein-binding sites in wells were coated with 1% bovine serum albumin (BSA; Sigma-Aldrich, San Luis, MO, USA) in PBS for 10 min, followed by three washes with PBST. Then, the plates were incubated for 1 h with 100 µL of patient serum (all analyzed sera were diluted in triplicate at 1:100 with PBS obtaining reproducible results in each assay). Policlonal serum (diluted 1:100) containing antibodies against Maize zein were produced in rabbits as previously described [22] and used as positive controls The plates were washed three times with PBST and incubated for 1 h with horseradish peroxidase (HRP)-conjugated anti-IgA antibodies (Sigma-Aldrich, San Luis, MO, USA) 1:3000 dilution in PBS) or HRP-conjugated anti-human IgG (Sigma-Aldrich, San Luis, MO, USA), 1:60,000 dilution in PBS). After three washes, the HRP activity was developed with 2,2′-azino-bis(3-ethylbenzothiazoline-6-sulfonic acid) (Sigma-Aldrich, San Luis, MO, USA). After 15 min, the optical density (OD) was read at 405 nm with a reference filter at 650 nm using a microplate reader (Awareness Technology Inc., Palm City, FL, USA). As a background control, we subtracted from each OD of serum samples the OD obtained when the ELISA wells contained no antigen but contained BSA as a blocking molecule. The cut-off values and index values for both IgA and IgG AZA were calculated as previously described [23]. The serum reactivity of IgA anti-zeins was expressed as an index value; that is, the optical density of the test serum divided by the cutoff value. The cutoff value was the mean + 2 SD of the absorbance values of 280 negative sera of healthy donors. Index values of 1.0 and above were considered to be positive.

### 2.4. Statistical Analysis

All the statistical analyses and graphic representations were performed using GraphPad Prism version 8 (San Diego, CA, USA). The normality of the data distribution was tested by the Kolmogorov–Smirnov test. The prevalence was compared through Fisher’s exact test, the odds ratios (OR), and the 95% confidence intervals (95% CI). Since the data were not normally distributed, continuous variables are described as medians (range), whereas categorical variables are expressed as percentages. Differences between groups were compared using the nonparametric, two-tailed Mann–Whitney or Kruskal–Wallis test, and all pairs of groups were compared by Dunn’s post-test. To determine positive associations between different variables, a two-tailed Spearman’s correlation test was used. Statistical significance was set at a *p* value of < 0.05.

## 3. Results

While gliadin, the major component of wheat gluten, is the most immunogenic prolamin in wheat and widely studied in terms of CD and IBS immunopathology and seroprevalence studies, little is known about the immune response and the prevalence of specific antibodies to prolamins in maize (zeins), a very important and a common dietary cereal in Latin American countries, which require further investigation regarding their potential as food immunogenic antigens in some bowel inflammatory disorders. First of all, we recruited a total of 37 subjects with CD, 375 subjects with IBS, and 302 healthy controls, and all the subjects were evaluated for anti-zein antibodies (AZA). Among CD subjects, the most common clinical manifestations were: abdominal bloating (81%), abdominal pain (72%), diarrhea (64%) and iron deficiency anemia (40%). Table 1 summarized the clinical and demographic characteristics of the evaluated subjects. The entire population was classified as Hispanic (Mexican Mestizos) and did not include any other race. None of the HC or IBS subjects were relatives for CD subjects.

### 3.1. Higher Levels of IgA AZA Antibodies in Celiac Disease than Irritable Bowel Syndrome

**Figure 1:**
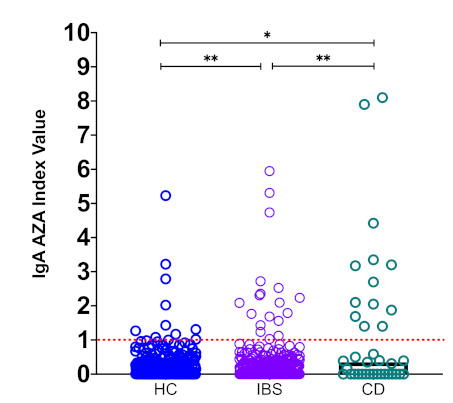
High serum levels of IgA anti-zein antibodies (AZA) in celiac disease (CD) patients. Cut-off values were calculated and determined as described in the Materials and Methods, and they are shown by dotted lines. Medians values are compared between the groups. * *p* <0.05, ** *p* <0.001.

In our patient cohort, the serum IgA AZA levels were significantly higher in patients with CD (median, 0.35 (range, 0–8.1); mean, 1.25) than in IBS patients (median, 0.0 (range, 0.0–5.94); mean, 0.24; *p* < 0.001) or HCs (median, 0.03 (range, 0.0–5.23); mean, 0.22; *p* < 0.05; Figure 1). Additionally, significant differences in the serum IgA AZA levels were found between IBS patients and HCs (*p* < 0.01).

**Figure 2:**
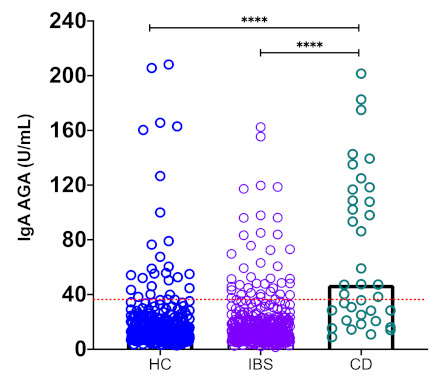
High serum levels of IgA anti-gliadin (AGA) in celiac disease (CD) patients. Cut-off values were calculated and determined as described in the Materials and Methods, and they are shown by dotted lines. Medians values are compared between the groups. **** *p* <0.0001

Next, we compared the serum levels of IgA antibodies against native gliadin (IgA AGA) in patients with CD, patients with IBS, and the HCs (Figure 2). Just like for the IgA AZA, the serum IgA AGA levels were statistically significantly higher in the CD patients (CD median 47.15 (range, 8.87–201.54); mean 69.2) versus the IBS patients (IBS median 12.44 (range, 1.53–162.42); mean 19.05; *p* < 0.0001) and the HCs (HC median 12.09 (range, 2.75–208.18); mean 19.4; *p* < 0.0001) There were no differences in the serum levels of IgA AGA between the IBS patients and the HCs (*p* > 0.05).

Interestingly, we did not detect IgG AZA antibodies in any patients from the CD, IBS, or HC groups, even in the IgA AZA-positive patients.

### 3.2. Higher Prevalence of Anti-Zein IgA (IgA AZA) Antibodies in Celiac Disease Patients than in Irritable Bowel Syndrome Patients and Healthy Controls

Given our previous results showing the higher serum levels of specific IgA AZA in the CD group, next we studied the prevalence of IgA AZA antibodies in CD and IBS patients compared to the HC group. In CD patients, thirteen out of thirty-seven patients were found to be IgA AZA-positive (35.1%). In the IBS group, sixteen (4.3%) out of three hundred and seventy-five patients tested positive for IgA AZA. Comparing the CD group against the IBS patients (35.1% CD vs. 4.3% IBS; *p* < 0.0001) and HC groups (35.1% CD vs. 2.3% HC; *p* < 0.0001), the CD patients showed the highest and statistically significant prevalence of IgA AZA antibodies. We did not find a statistically significant difference in the prevalence of IgA AZA in the IBS and HC groups (4.3% IBS vs. 2.3% HC; *p* = 0.0726). A further analysis in the base of the clinical IBS subtype also revealed no difference between the subtypes and the HC group (*p* > 0.05) (Table 2).

### 3.3. Higher Prevalence of Anti-Gliadin IgA (IgA AGA) Antibodies in Celiac Disease Patients than Irritable Bowel Syndrome Patients and Healthy Controls

Next, we studied the prevalence of anti-Gliadin IgA (AGA) antibodies in all the studied subjects. As in the previous results for IgA AZA prevalence, the CD patients also showed a higher IgA AGA prevalence, which was significantly different to the observed prevalence in the IBS patients (64.9 % CD vs. 17.7 % IBS; *p* < 0.0001) and the HCs (64.9% CD group vs. 12.3% HC group; *p* < 0.0001). We did not find any difference in the prevalence of IgA AGA between the IBS patients or IBS subtypes with the HC group (*p* > 0.05) (Table 2).

### 3.4. High Serum Levels of Celiac Disease-Related Antibodies in the IgA AZA-Positive Patients

We decided to analyze in the celiac disease group a potential association between the presence of IgA AZA antibodies and the serum levels of celiac disease-related IgA antibodies: anti-tissue transglutaminase IgA antibodies (tTG), anti-gliadin IgA antibodies (AGA IgA), or anti-deaminated gliadin peptide IgA antibodies (DPG IgA). We first divided the celiac disease patients into two groups—IgA AZA positive or negative—and then we compared both groups for the mean of the serum levels for each celiac disease-specific IgA antibody (Table 3). The IgA AZA-positive CD patient group showed higher serum levels of IgA DGP (mean = 51.5 U/mL; *p* = 0.0337) and IgA AGA (mean = 97.99 U/mL; *p* = 0.0359) than the IgA AZA-negative group: IgA DGP (mean = 29.88 U/mL) and IgA AGA (mean = 56.37 U/mL). No differences were found when we compared the serum levels of IgA tTG between both the positive and negative IgA AZA patients.

### 3.5. Correlation Analysis of IgA AZA, IgA DGP, and IgA AGA Serum Levels in Celiac Disease and Irritable Bowel Syndrome

**Figure 3:**
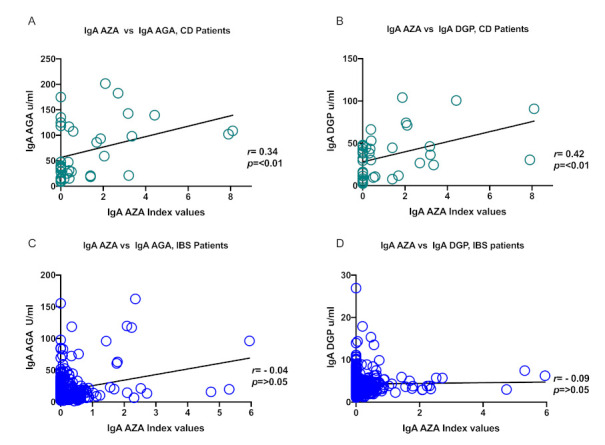
Correlation analysis between IgA AZA and IgA AGA in CD and IBS patients. (**A**) IgA AZA vs. IgA AGA serum levels in celiac disease patients, (**B**) IgA AZA vs. IgA DGP serum levels in celiac disease patients, (**C**) IgA AZA vs. IgA AGA serum levels in IBS patients, (**D**) IgA AZA vs. IgA DGP serum levels in IBS patients.

Given our previous results, we decided next to perform a correlation analysis between all the serological markers in both patient groups: celiac disease and irritable bowel syndrome. A significant positive correlation was found between IgA AZA with IgA AGA (*r* = 0.34, *p* < 0.01) and IgA DGP (*r* = 0.42, *p* < 0.001; Figure 3a,b) in the celiac disease group. No statistically significant correlation was found between the levels of IgA AZA and IgA tTG in the CD patients (data not shown). Additionally, no statistically significant correlation was found between the levels of IgA AZA and IgA AGA (*r* = −0.04, *p* > 0.05) or IgA DGP (*r* = −0.09, *p* > 0.05; Figure 3b,c) in patients with irritable bowel syndrome.

## 4. Discussion

Celiac disease (CD) and irritable bowel syndrome (IBS) are two different chronic inflammatory conditions affecting the gastrointestinal tract with similar and overlapping symptoms, which can result in the symptoms of CD patients often being confused with the symptoms of IBS, which makes diagnosing celiac disease that much more difficult. In contrast with IBS, a common multifactorial disorder that affects the large intestine, CD is an autoimmune disorder that occurs in genetically predisposed subjects and is triggered by a diet rich in cereals such as wheat, barley, and rye, which induce chronic inflammation of the proximal small intestine, resulting in villous atrophy and malabsorption [24]. The standard treatment for celiac disease is the maintenance of a gluten-free diet [25], and there are numerous grains with which to substitute wheat in the CD patient diet, including maize, one of most commonly consumed cereals in the world [26,27]. Maize has been considered for a long time as a non-toxic cereal for celiac disease patients, and the detrimental effect of this cereal in the CD has not been well studied, either in the persistence of the symptoms or in the presence of refractory disease. Thus, this remains a controversial issue, and follow-up studies on the long-term effects of zein are needed. Previous studies have suggested before that maize zeins and some zein-derived peptides could be immunogenic in some in vitro, ex vivo, and in vivo experimental approaches. In vitro*,* zein peptides induce increased levels of IFN-γ in intestinal biopsies derived from celiac disease patients. The in vitro stimulation of the intestinal CACO-2 cell line with two zein fractions (3–5 and 1–3 kDa) induced the production/activation of innate molecules such as IL-8, p38 MAPK, and COX2 and the release of ZO-1 [28]. It has been hypothesized that maize prolamins could induce also a T cell response in some celiac disease patients [29]. In vivo, adverse responses have been reported to maize proteins after oral challenge in some CD patients [30]. All this previous scientific evidence suggests that maize prolamins could be an immunogenic dietary antigen with important implications in the immunopathophysiology of some chronic inflammatory bowel disorders. Here, we studied the humoral immune response to maize zein, analyzing the serum levels and prevalence of IgA and IgG AZA antibodies in two different inflammatory bowel diseases: celiac disease and irritable bowel syndrome. In spite of the fact that both diseases share a chronic inflammatory condition as the hallmark of their clinical course, we found higher levels and a higher prevalence of IgA AZA antibodies in the CD patient group compared to in IBS patients and healthy controls, suggesting that the genetic susceptibility in CD influences the immunological response to other dietary prolamins besides gliadin in some celiac patients. In agreement with our results showing a high prevalence of IgA antibodies to maize zein, a humoral immune response to maize prolamins has been described before, characterized mainly by humoral IgA immunoreactivity. Maize prolamins deamidated by TG in vitro are recognized by purified IgA from some CD patients’ sera [31]. Additionally, IgA antibodies against maize prolamins were detected in several CD patients. Our results showing high levels and a high prevalence of anti-zein IgA antibodies in CD patients open two different and interesting possibilities to explore in this particular disease: a potential cross-reactivity phenomenon between gliadin and zein or a specific immune response against maize zeins in genetically susceptible individuals. A previous study of Skerrit and Cols has shown before in CD patients the presence of a low IgA level and even very low levels of IgG antibodies to maize proteins, with no cross-reactivity with gliadin [32]. In contrast to the Skerrit and Cols’ findings, we observed in high levels of IgA but an absence of IgG AZA antibodies our CD patients. Our observation about the absence of IgG AZA antibodies was extended to the IBS and HC groups. The lack of an IgG AZA antibody response in all the studied groups was a striking result in our study, in contrast with the widely diversified humoral immune response to gliadin characterized by the production of both anti-gliadin IgA and IgG isotypes. AGA antibodies are not CD specific because they have lost much of their diagnostic significance in recent years due to the introduction of the more sensitive and specific tests, [33]. Celiac disease is associated with a variety of IgA and IgG antibodies against native and deamidated gliadin and some autoantibodies, including endomysial and tissue transglutaminase (tTG) antibodies [34]. The IgA isotype of these antibodies usually predominates in celiac disease, but individuals may also produce IgG isotypes, particularly if the individual is IgA-deficient [35]. Positive test results for deamidated gliadin antibodies, IgA or IgG, are consistent with the diagnosis of celiac disease [36]. The humoral immune response in CD patients against other prolamins besides gliadin has been described, particularly for oat. In previous clinical studies, an anti-avenin antibody response has been shown in celiac children on a gluten-free diet with or without oats. Celiac children had significantly higher levels of antibodies (IgG and IgA) to both avenin and gliadin compared to a control children group [37]. Interestingly, those previous studies have shown no cross-reactivity between anti-avenin and antigliadin antibodies. Another important conclusion was derived from our study: the observed missing IgG immune response to maize zein in the CD, IBS, and healthy subjects confirmed the absence of a specific anti-zein IgG response or a potential cross-reaction between specific anti-gliadin IgG antibodies against native zein. The presence of anti-zein IgA but not IgG antibodies in the CD, IBS, and even healthy control groups could be explained by the differential exposure and access of the zein antigen to the mucosal versus systemic immunological environment. Normally, IgA antibodies are produced by B cells in local lymph nodes in the mucosa, reflecting a local response. IgG antibodies, on the other hand, describe a systemic exposure and systemic response. Therefore, the presence of specific IgG antibodies directed against food antigens reflects systemic reactions to antigens penetrating due to damage to the epithelial barrier. Finally, in our study we observed in the CD patients a positive correlation between IgA AZA antibodies and IgA AGA and IgA DPG; the last one is an important serological biomarker with a high sensitivity and specificity in untreated, biopsy-proven celiac disease and is comparable also with the tissue transglutaminase (tTG) IgA antibody test [38].

## 5. Conclusion

Our study had some limitations that should be addressed in the future. First of all, the number of CD subjects was small compared to the IBS and the healthy control groups. It is important to mention that diagnosis of CD in Latin America as in other parts of the world represents a real challenge, because the disease is underdiagnosed. The other limitation was that just 16 subjects agreed to undergo duodenal biopsies; thus, the remaining 21 subjects were diagnosed in base of specific antibodies for CD and they were classified as subjects with celiac autoimmunity. However, some guidelines and recent studies have considered recommended a no-biopsy approach for the diagnosis of adult CD, especially if high IgA tTG titres (≥10 times the upper limit of normal ULN) are found [39]. At this time, we did not perform a clinical correlation with symptoms or disease activity, and no HLA typing was performed either; thus, further studies are required. Future prospective clinical studies are needed to evaluate AZA-IgA immunological relevance as a biomarker in association to the disease activity, in the follow-up of CD patients following a gluten-free diet and in vitro experiments to obtain mechanistic insights about the role of AZA IgA antibodies.

## Figures and Tables

**Table 1:** Demographics and clinical characteristics of patients and healthy controls.

Diagnosis	*n*	Sex (F/M)	Age (years) Median (range)
**Celiac disease**	37	28/9	45 (18–85)
**IBS with constipation**	121	107/14	43 (18–80)
**IBS with mixed pattern**	210	173/37	41.5 (12–77)
**IBS with diarrhea**	44	40/4	45 (18–69)
**Total IBS**	375	320/55	43 (12–80)
**Healthy controls**	302	167/135	21 (17–51)

IBS: irritable bowel syndrome.

**Table 2:** Prevalence of anti-zein IgA and anti-gliadin IgA antibodies in celiac disease, irritable bowel syndrome, and healthy controls.

Diagnosis	*n*	IgA AZA^+^ *n* (F/M)	Prevalence (%)	*p*-Value	IgA AGA^+^ *n* (F/M)	Prevalence (%)	*p*-Value
CD	37	13 (8/5)	35.1	<0.0001	24 (16/8)	64.9	<0.0001
IBS-C	121	4 (4/0)	3.3	0.5185	15 (12/3)	12.4	1.0000
IBS-M	210	12 (12/0)	5.7	0.0571	32 (29/3)	15.2	0.3582
**IBS-D**	44	0 (0/0)	0	1.0000	8 (8/0)	18.2	0.3342
**Total IBS**	375	16 (16/0)	4.36	0.0726	55 (49/6)	17.7	0.3697
**HC**	302	7 (3/4)	2.3	–	37 (11/26)	12.3	---

IgA AZA^+^, positive anti-zein IgA (Immunoglobulin A) antibodies; IgA AGA^+^, positive anti-gliadin IgA (immunoglobulin A) antibodies; CD, celiac disease; IBS, irritable bowel syndrome; IBS-C, IBS with constipation; IBS-M, IBS with mixed pattern; IBS-D, IBS with diarrhea; HC, healthy controls.

**Table 3:** Comparison of serological markers levels (U/mL) in patients with celiac disease in the IgA AZA-positive or -negative groups.

Serological Marker		IgA AZA Negative (*n* = 24)	IgA AZA Positive (*n* = 13)	*p-*Value
IgA tTG	*n* (%)	15 (62.5)	7 (53.9)	0.7304
	U/mL	36.1 (2.9–133)	30.9 (6.9–99.8)	0.8839
IgA DGP	*n* (%)	12 (50.0)	9 (69.2)	0.3149
	U/mL	29.88 (4.05–66.3)	51.5 (7.6–104.1)	0.0337
IgA AGA	*n* (%)	14 (58.3)	10 (76.9)	0.3052
	U/mL	56.37 (8.87–135.14)	97.99 (18.3–201.5)	0.0359

IgA, immunoglobulin A or G; AZA, anti-zein antibodies; tTG, anti-tissue transglutaminase antibodies; DGP, anti-deaminated gliadin peptide antibodies; AGA, anti-native gliadin antibodies.
